# How to Form Wellbeing Perception and Its Outcomes in the Context of Elderly Tourism: Moderating Role of Tour Guide Services

**DOI:** 10.3390/ijerph17031029

**Published:** 2020-02-06

**Authors:** Jinsoo Hwang, Jinkyung Jenny Kim, Jenni Soo-Hee Lee, Noman Sahito

**Affiliations:** 1 The College of Hospitality and Tourism Management, Sejong University, 98 Gunja-Dong, Gwanjin-Gu, Seoul 143-747, Korea; jhwang@sejong.ac.kr; 2 School of Hotel and Tourism Management, Youngsan University, 142 BansongBeltway, Haeundae-Gu, Busan 48015, Korea; jennykim1120@gmail.com; 3 Department of City & Regional Planning, Mehran University of Engineering & Technology, Jamshoro 76062, Pakistan; nauman.sahito@faculty.muet.edu.pk

**Keywords:** quality of life, tour guide, brand prestige, senior tourists, word-of-mouth

## Abstract

Many people travel to improve their wellbeing perception (WBP), and senior tourists in South Korea are no exception in that they hope to enhance their quality of life through tourism. As such, this study explored the significance of WBP in the senior tourism industry in South Korea. The current paper collected samples from 349 senior tourists. Analysis of data indicated that brand prestige contributes to increasing WBP among seniors and improving consumer attitude. In addition, it was found that WBP positively affects both consumer attitude and word-of-mouth (WOM). Lastly, tour guide services moderated the relationship between (1) brand prestige and WBP and (2) consumer attitude and WOM. The current paper then presents theoretical and practical implications of the statistical results.

## 1. Introduction

South Korea is one of the fastest aging countries. For example, about 14% of the total population in 2016 was over the age of 65 [1]. Furthermore, the agency reported that the ratio of older people is expected to comprise 25% of South Korea’s population by 2030 and 40% by 2060. Based on these statistics, it can be assumed that the number of elderly tourists also increases, and as such, there is a growing interest in senior tourism in South Korea. For instance, the government provides travel and cultural education programs to encourage the elderly to travel independently [2].

In modern society, people consider quality of life (hereafter QOL) in terms of material abundance and mental relaxation. Hence, many scholars have identified the concept of wellbeing perception (hereafter WBP) in diverse fields (e.g., businesses, psychology, and sociology) [3,4]. The concept of WBP is important to senior tourism because older citizens have been shown to want to take trips to meet the needs of their wellbeing [5,6]. In other words, the elderly’s desire to increase their QOL through travel. For this reason, this study examines how to generate WBP and its outcomes in senior tourism.

It has also been shown that tour guide services are a critical factor in package tourists’ satisfaction [7,8] and play a significant role in the formation of word-of-mouth (hereafter WOM) and revisit intention (hereafter RI) [9]. For senior tourists, the role of a tour guide is more important than for other tourists since they may experience difficulties, such as physical weakness, during a trip. Consequently, senior tourists tend to rely more heavily on their tour guide during travel [10]. Pereira [11] also has argued that, although tourists may be satisfied with a tourist destination, they will not have a good impression if the tour guide does not perform their role properly. These arguments suggest a moderating role for tour guide services since travelers’ satisfaction can be determined by the quality of tour guide services. Despite the significance of tour guide services in the senior tourism industry, relatively little research has focused on it.

Therefore, the current research attempted to fill this gap by empirically investigating the antecedents and consequences of WBP with the moderating role of the tour guide services in senior tourism. More specifically, the objectives of this research were to examine: (1) The influences of brand prestige on WBP, consumer attitude, and WOM; (2) the effects of WBP on consumer attitude and WOM; (3) the relationship between consumer attitude and WOM; and (4) the moderating effect of tour guide services in the proposed model.

## 2. Literature Review

### 2.1. Wellbeing Perception (WBP)

WBP has been studied for a long time in various fields. It can be defined as the extent to which a certain consumer good/service provides the overall perception of the QOL [12]. In other words, the level of QOL is a crucial standard in determining WBP [13]. For instance, if senior tourists perceive that their QOL is increased, then they have high levels of WBP. More importantly, WBP has a significant influence on consumer behavioral intentions (hereafter BI) [14,15], suggesting that when consumers feel that a certain product/service satisfies their WBP needs, they tend to purchase the product/service. For this reason, many studies have consistently tried to focus on how to form BI using the concept of WBP.

For instance, Kim, et al. [16] investigated how to form BI using 433 chain restaurant patrons. They found that WBP played a crucial role in the formation of BI. Lin [14] explored the influence of WBP on RI in the context of hot springs. They analyzed 524 Taiwanese tourists and suggested that WBP is a significant predictor of RI. Hwang and Han [3] also explored the importance of WBP using 330 luxury cruise passengers. They showed that when passengers feel that their QOL is enhanced, they would take the cruise in the future. Ahn et al. [17] proposed a theoretical model in order to find the relationship between WBP and BI using 205 first-class passengers. They suggested that when customers have a high level of WBP, they are willing to take first-class flights in the future. Hwang and Lyu [18] investigated the relationship between WBP and BI using 230 golf tournament tourists. They argued that WBP is a significant factor influencing RI.

### 2.2. Brand Prestige

As brand prestige has received much attention in the hospitality and tourism industry including luxury cruise, first-class flights, private country clubs, and casinos, it has been studied by many scholars [3,17,19,20]. The concept of brand prestige is defined as a relatively higher status of products/services positioning related to a brand when compared with other competing brands [21,22]. The criteria for evaluating the level of brand prestige can be an intrinsic and exclusive ‘know-how’ that relates with a certain attribute of a product/service [23]. In addition, consumers are more likely to buy a prestigious brand to show that they belong to a special group unlike other consumers [22]. They are also called prestige brand seekers. More importantly, brand prestige plays a crucial role in forming brand loyalty [3,24]. For instance, Hwang and Han [3] found that when cruise passengers have high levels of brand prestige, they are more likely to have brand loyalty. In addition, Jin, Line, and Merkebu [24] also identified that brand prestige is a crucial predictor of brand loyalty.

The current paper proposed the relationship between brand prestige and WBP according to the following theoretical and empirical backgrounds. Increasing the QOL is critical to the perception of wellbeing [12]. In particular, consumers try to buy a prestigious brand to enhance their QOL [25], indicating that brand prestige is a critical antecedent of WBP. For instance, when the elderly feel that the package tour had high status, they tend to perceive high levels of wellbeing. Previous studies also suggested that brand prestige helps to increase WBP. For example, Hwang and Han [3] tried to explore how brand prestige affects WBP using 330 passengers. The authors suggested that brand prestige is an important factor in forming WBP. In addition, Hwang and Hyun also suggested that brand prestige is an important factor affecting WBP in the restaurant industry. Therefore, the current paper proposed the following hypothesis:

**Hypothesis 1** **(H1).** *Brand prestige is a critical factor in forming WBP.*

### 2.3. Consumer Attitude

The concept of consumer attitudes is defined as a feeling or opinion toward a certain product or service [26,27]. Consumer attitudes are very important for companies to take a competitive advantage, so many existing studies have examined consumer attitudes in diverse fields [28,29]. In addition, it is required to distinguish between customer satisfaction and consumer attitude. It is generally accepted that customer satisfaction is an important criterion in evaluating product/service performance [30,31]. However, customer satisfaction measures the performance of a certain product/service provided by a brand, while customer attitudes evaluate the overall performance of a brand [28,32]. In other words, the concept of consumer attitudes is greater than the concept of customer satisfaction. More importantly, consumer attitudes play a crucial role in creating BI such as RIs and WOM, so it is important for companies to understand consumer attitudes [33,34].

According to Steenkamp, Batra, and Alden [22], consumers tend to purchase prestigious brands in order to match their image and the prestigious brands. Similarly, Bizman and Yinon [35] suggested that consumers hope to link their image prestigious brands by purchasing such brands. Therefore, if a particular brand helps the consumer’s image to be prestigious, they generally have a favorable attitude toward that brand. Based on this logic, the following hypothesis is suggested:

**Hypothesis 2** **(H2).** *Brand prestige is a critical factor in forming consumer attitude.*

Consumers buy prestigious products by considering their self-image, which leads to increase their QOL [3,23]. In particular, such a significance of QOL leads to a positive attitude toward the product [36,37]. Thus, when tourists perceive a high level of wellbeing during the tour, they tend to have favorable attitudes toward the travel company brand. Prior research also confirmed the argument. For example, Hwang and Han [3] found that when luxury cruise passengers perceive that the luxury cruise satisfies their WBP needs, they have a good feeling for the brand. Then, Hwang and Hyun [38] suggested that when first-class airline travelers have a high level of WBP, they would have positive emotions toward the brand. Thus, we proposed the following hypothesis:

**Hypothesis 3** **(H3).** *Seniors’ WBP is a critical factor in forming consumer attitude.*

### 2.4. WOM

According to Westbrook [39], WOM is defined “informal communication directed at other consumers about the ownership, usage or characteristics of particular goods and services and/or their sellers.” Consumers tend to tell others, such as family, relatives, and friends, about their experiences after using a particular product/service [40,41]. The influence of word of mouth is greater than commercial advertisement because in terms of word of mouth, people get information from acquaintances [42,43], so WOM acts as an advertisement in which consumers voluntarily promote products/services. In addition, there are two types of WOM, positive and negative. Positive WOM occurs about three times more frequently than negative [44].

Many previous studies have empirically supported the influence of brand prestige on WOM. For example, Wong and Zhou [45] showed that brand prestige plays a crucial role in enhancing WOM. Furthermore, Hwang and Han [3] suggested that brand prestige was a key factor influencing brand loyalty in the luxury cruise industry. Hwang, Han, and Choo [20] also found that when consumers have high levels of brand prestige from a private country club, they try to spread the news about the good aspects of the club to others. Hence, the current paper proposed a relationship between brand prestige and WOM:

**Hypothesis 4** **(H4).** *Brand prestige is a critical factor in forming WOM.*

Additionally, this research proposed the influence of WBP on brand loyalty. Kim, Mi Jeon, and Sean Hyun [16] confirmed that WBP helps to enhance brand loyalty in the restaurant industry. Additionally, Hwang and Lyu [18] confirmed that WBP is a critical antecedent of loyalty in the context of golf tournament. Furthermore, Hwang and Hyun [38] identified the effect of WBP on loyalty in the context of first class flights. Thus, when senior tourists have a high level of WBP during a tour, they will say positive things about the travel agency to others:

**Hypothesis 5** **(H5).** *Seniors’ WBP is a critical factor in forming WOM.*

Prior research has suggested that consumer attitude positively affects WOM. For instance, Spector and Fox [46] indicated that if a customer has a good attitude toward a particular brand, he/she has a high level of BI. Han and Hyun [47] also confirmed the influence of consumer attitude on BI. In addition, Han et al. [48] suggested that there is positive relationship between consumer attitude and BI. More recently, Trang et al. [49] found that consumer attitude is a crucial factor affecting BI. Based on these arguments, the current paper proposed the following hypothesis:

**Hypothesis 6** **(H6).** *Consumer attitude is a critical factor in forming WOM.*

### 2.5. The Moderating of Tour Guide Services

The tour guide has many roles during package travel, so he/she is known as information givers [50,51]. Tour guides can be defined as people who guide individuals or groups on visits around the buildings and landscapes of region or city [52]. For elderly tourists, the tour guide has more special meaning. An elderly tourist is more dependent on tour guides because they are physically weaker and less capable of searching for information than younger tourists [10]. More importantly, tour guide services have a very important effect on travel satisfaction [7]. That is, even though tourists are satisfied with a travel, if his/her services do not meet the needs of tourists, the overall satisfaction with the trip is not high. In addition, overall satisfaction helps to enhance BI, such as RIs and WOM [53,54], so travel agencies should thoroughly manage the tour guide through a systematic system. It is widely accepted that the performance of a tour guide is significant for the tour package because he/she acts as an interface between tourists and tourist destinations [55]. That is, even if the tourist destinations are very satisfactory, tourists’ overall satisfaction will not be high unless the tour guides perform their role well. Chang [9] also suggested that the tour guide performance is the key factor affecting tourists’ overall satisfaction, which enhances RIs. Additionally, Huang, Hsu, and Chan [7] argued that the destinations as well as the overall image of the travel agency can vary depending on the tour guide performances. Therefore, the current paper suggested moderating effects of tour guide services in the senior tourism industry:

**H7a.**  *Tour guide services moderate the relationship between brand prestige and seniors’ WBP.*

**H7b.**  *Tour guide services moderate the relationship between brand prestige and consumer attitude.*

**H7c.**  *Tour guide services moderate the relationship between seniors’ WBP and consumer attitude.*

**H7d.**  *Tour guide services moderate the relationship between brand prestige and WOM.*

**H7e.**  *Tour guide services moderate the relationship between seniors’ WBP and WOM.*

**H7f.**  *Tour guide services moderate the relationship between consumer attitude and WOM.*

### 2.6. Proposed Conceptual Model

**Figure 1:**
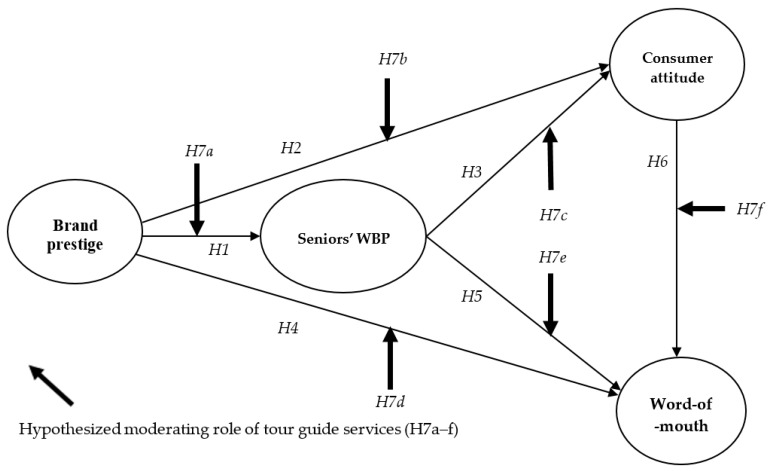
Proposed conceptual model.

According to the theoretical background mentioned above, the current paper presents the research model in Figure 1.

## 3. Materials and Methods 

### 3.1. Measurement

This paper measured five constructs: Brand prestige, seniors’ WBP, consumer attitude, WOM, and tour guide services. First, brand prestige was measured with three items employed by [19,56]. Second, seniors’ WBP was measured with three items used by [12,57]. Third, consumer attitude was measured with five items adapted by [47,58]. Fourth, WOM was measured using three items [59,60]. Fifth, tour guide services were measured with three items employed by [61,62]. The questions were asked using a 7-point Likert-type scale. 

### 3.2. Data Collection

In this study, data were collected in Seoul, South Korea, using face-to-face survey. The sample was the elderly in this study, so we conducted the survey at five famous parks where the elderly frequently visit. Since the legal standard of the elderly in South Korea is 65 years old, the questionnaire was started after interviewers confirmed that respondents were over 65 years old before the survey. The interviewers had a good understanding of the study because they have been fully educated about the study before the survey. In addition, respondents were excluded from the questionnaire if they had not traveled abroad in the last six months. A questionnaire was distributed to 400 people and 349 people completed the questionnaire. Among them, 24 people were deleted because of visual inspection and a Mahalanobis distance check. As a result, 325 usable responses were employed in the further analysis.

## 4. Data Analysis

### 4.1. Descriptive Statistics

Table 1 describes the respondents’ demographic profile. Of 325, 29.2% were women and 70.2% were men. The mean age of the sample was 69.20 years, ranging from 65 to 80. The majority of respondents had bachelor’s or graduate degrees (55.7%, *n* = 181), followed by high school diploma (22.5%, *n* = 73). The majority of respondents were married (96.6%, *n* = 314). With regard to monthly income, 21.8% (*n* = 71) reported that their annual income was between US$ 3,001~US$ 4,000.

### 4.2. Confirmatory Factor Analysis

In order to evaluate the uni-dimensionality of the constructs and validate the measurement model, confirmatory factor analysis (hereafter CFA) was performed [63,64]. The results of CFA showed an acceptable model fit (χ^2^ = 259.722, *df* = 71, *p* < 0.001, χ^2^/*df* = 3.658, NFI = 0.957, CFI = 0.968, IFI = 0.968, TLI = 0.959, RMSEA = 0.071) [65]. All of the factor loading values in our research were greater than 0.897, all of which were significant at the *p* < 0.001 level. It also provided evidence of convergent validity since all the loadings were greater than 0.70 [66,67]. Table 2 indicated the specific items of theoretical constructs employed in this study, together with their standardized factor loadings.

As shown in Table 3, the average value of brand prestige was 4.30 and the standard deviation was 0.94. In terms of seniors’ WBP, the average value was 4.37 and the standard deviation was 0.95. In the case of consumer attitude, the average value was 4.52 and the standard deviation was 1.10. Lastly, the average value of WOM was 4.40 and the standard deviation was 0.99. In addition, the values of average variance extracted (AVE) were higher than the 0.50 standard [68,69] for all of the proposed constructs. Among them, the AVE value for WOM was the highest at 0.859. Based on the high level of factor loadings and the AVE values, the convergent validity for the scale items was well achieved [70]. In Table 4, all *R*^2^ between a pair of constructs are lower than the values of AVE for each construct, indicating satisfactory discriminant validity. Lastly, the internal consistency was proved employing composite reliabilities of each construct. Hair et al. [71] and Asif et al. [72] recommended a standard of 0.70, and all of the composite reliabilities exceeded 0.70, suggesting strong internal consistency. 

### 4.3. Structural Model

The proposed model with four constructs was estimated using structural equation modeling (hereafter SEM) analysis [73]. Fit indices provided by SEM analysis showed that the model had an adequate fit (χ^2^ = 259.722, *df* = 71, *p* < 0.001, χ^2^/*df* = 3.658, NFI = 0.957, CFI = 0.968, IFI = 0.968, TLI = 0.959, RMSEA = 0.070) [65,74]. In Table 4, Hypotheses 1, 2, 4, 5, and 6 were statistically supported, while Hypothesis 3 was not supported. More specifically, Hypothesis 1, which proposed the effect of brand prestige on seniors’ WBP, was statistically supported (*β* = 0.896, *p* < 0.05). There was a positive relationship between brand prestige and consumer attitude (*β* = 0.371, *p* < 0.05), which supported Hypothesis 2. However, there was no relationship between brand prestige and WOM (*β* = 0.102, *p* > 0.05). In addition, seniors’ WBP played an important role in the formation of consumer attitude (*β* = 0.418, *p* < 0.05) and WOM (*β* = 0.255, *p* < 0.05). Hence, Hypotheses 4 and 5 were supported. Lastly, consumer attitudes positively affected WOM (*β* = 0.465, *p* < 0.05), so hypothesis 6 was supported. Moreover, the values of t-statistics were also greater than 1.96 [75], which provides evidence of significance.

### 4.4. Moderating Role of Tour Guide Services

**Figure 2:**
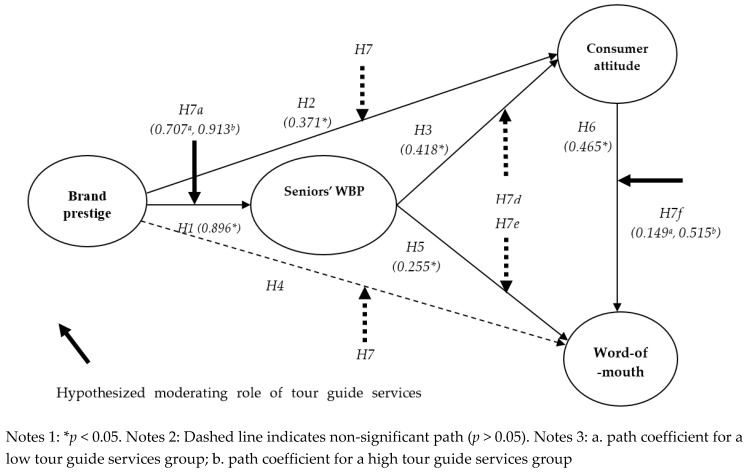
Structural model results.

Figure 2 shows the results of the moderating role of tour guide services. To test the moderating role of tour guide services, multiple-group analyses were employed [65]. Respondents (*n* = 325) were divided into two groups (a low tour guide services group = 133 and a high tour guide services group = 192) based on a moderator score [76]. First, the moderating effect of tour guide services in the relation between brand prestige and seniors’ WBP was then evaluated (H7a). The chi-square difference between the constrained model and the unconstrained model was significant at the 0.05 level (χ^2^ = 6.593 > χ^2^_0.5_(1) = 3.84, df = 1), and Hypothesis 7a was supported. This finding indicates that the effect of brand prestige on seniors’ WBP was statistically different across tour guide services levels. With regard to the low tour guide services group, the path coefficient between brand prestige and seniors’ WBP was 0.707 (*p* < 0.05). In contrast, for the high tour guide services group, the path coefficient was 0.913 (*p* < 0.05). 

Second, the moderating effect of tour guide services in the relation between brand prestige and consumer attitude was evaluated (H7b). The chi-square difference between the two constructs was not significant at the 0.05 level (χ^2^ = 3.051 < χ^2^_0.5_(1) = 3.84, df = 1). This finding means that the influence of brand prestige on consumer attitude is not significantly different across tour guide services levels, and Hypothesis H7b was not supported. 

Third, the moderating effect of tour guide services in the relation between seniors’ WBP and consumer attitude was assessed (H7d). The chi-square difference between the constrained model and the unconstrained model was not significant at the 0.05 level (χ^2^ = 1.900 < χ^2^_0.5_(1) = 3.84, df = 1). This result indicates that the effect of seniors’ WBP on consumer attitude is not significantly different across tour guide services levels, and Hypothesis 7d was not supported.

Fourth, the moderating role of tour guide services in the relationship between seniors’ WBP and WOM was evaluated (H7d). The chi-square difference between the two models was not significant at the 0.05 level (χ^2^ = 0.313 < χ^2^_0.5_(1) = 3.84, df = 1). This result shows that the effect of seniors’ WBP on WOM is not significantly different across tour guide services levels, and Hypothesis 7e was not supported.

Lastly, the moderating effect of tour guide services in the relationship between consumer attitude and WOM was also checked (H7f). The chi-square difference between the constrained model and the unconstrained model was significant at the 0.05 level (χ^2^ = 6.363 > χ^2^_0.5_(1) = 3.84, df = 1). Thus, Hypothesis 7f was supported. With regard to the low tour guide services group, the path coefficient between consumer attitude and WOM was 0.149 (*p* < 0.05). In contrast, for the high tour guide service group, the path coefficient was 0.515 (*p* < 0.05).

## 5. Discussions and Implications

One in five people will be aged over 60 years by the mid-21st century [77]. Together with the accelerated aging phenomenon, the growing attention on wellbeing is notable in today’s society and creates new opportunity in the tourism industry; specifically, package tour for elderly tourists [5,6,78,79,80]. The current research is an endeavor to fill the gap in the context of elderly tourism by focusing on WBP and tour guide services. The conceptual model was developed based on comprehensive literature review and empirical evidences. Then, SEM and multiple-group analyses based on 325 samples were conducted to examine the hypothesized associations among proposed variables. The results of data analyses successfully identified antecedents and consequences of WEP and impacts of tour guide services. Hence, our study contained the following originalities in theory and implications in practice. 

First, the present research incorporated WBP in the senior tourism into the theoretical framework in order to discover the antecedents and consequences through the inclusion of the moderating impact of tour guide services. In general, senior tourists are known to require extra care along with the higher spending and prolong length of stay, and thus a more comprehensive understanding is necessary in order to cater to the diversity of needs from senior tourists [81]. Despite the increased studies in parallel with the attention placed on the senior tourism, Patterson and Balderas [79] addressed insufficient importance given in current literature, and it resulted in market practitioners’ lack of understanding toward senior tourists. With this respect, this empirical study contributed to extend our knowledge on how WBP is formed and is associated with critical variables in predicting senior tourists’ responses. It also provided a new insight into the current knowledge of senior tourism by investigating the roles of tour guide services. 

Second, brand prestige (*β* = 0.896, *p* < 0.05) was verified as an essential antecedent of seniors’ WBP, and this is consistent with prior research [3,25]. Furthermore, the positive impact of brand prestige (*β* = 0.371, *p* < 0.05) on consumer attitude was confirmed. Brand prestige was regarded as a higher status of product or service itself, and the criteria for evaluating brand prestige was described as an intrinsic and exclusive value [22,23]. Therefore, travel agencies in the field of senior tourism should make long-term endeavors in creating the reputations that their tour packages have premium value and are upscale. In other words, continuous advertisements are recommended to position themselves as experts in the senior tourism and promote superior benefits provided so that the brand prestige is imprinted in potential senior consumers. Despite the inconsistency of defining senior (e.g., 65 years and older according to World Health Organization versus 55 years and older according to European Commission), there is a high possibility that digital channels might not be effective to seniors [80]. Hence, we inferred that offline channels are more preferred in this specific market and utilizing in-person sales activities are suggested. For example, conducting sales visits to the communities where seniors are generally gathered (e.g., senior citizens’ center) or placing a tourism desk at the home for the aged would be more strategic approaches. These examples will also help to create a senior-friendly image of travel agencies, which will enhance brand prestige and eventually enhance seniors’ WBP and the formation of attitude toward a particular travel agency. 

Third, seniors’ WBP was found to be a significant predictor of customers’ responses. Concretely, the association between WBP and attitude (*β* = 0.418, *p* < 0.05) and WBP and WOM (*β* = 0.255, *p* < 0.05) were statistically supported. Numerous scholars emphasized the importance of wellness in predicting customer behavior in the tourism context and our results supported the extant literature [12,16,37,38]. More importantly, senior tourists would like to ensure the travel experience meets their needs inducing physical and social satisfaction and increasing their QOL [80], and therefore a wide range of programs are suggested for variety appealing to different senior age groups’ interests. In this respect, wellness programs specializing in health and fitness can be composed with different levels and types of products. For instance, tour operators may consider package programs that include a meditation and various treatments enhancing mental and physical wellbeing such as aromatherapy or yoga. Alternatively, programs consisting of exploring luxury gastronomic experiences are proposed to build the impression that the package tour contributes to QOL. In addition, in consideration of the aging population all over the world, the roles of government and travel bureaus are necessary. As an example, Nella and Christou [81] introduced good practices of the globe, and one example is the Europe Senior Tourism (EST) program. It is an all-inclusive program, which is financially supported by local bureaus, and it would be meaningful to incorporate travel to build health societies. Moreover, since the senior tourism market is recognized as having less seasonality [80,81], it would also be beneficial for travel destinations to take the advantage of looking after senior tourists. 

Fourth, the impact of brand prestige on seniors’ WBP was significantly dissimilar (*β*_low group_ = 0.707, *p* < 0.05; *β*_high group_ = 0.913, *p* < 0.05) between the groups of people with low tour guide services and high tour guide services. More specifically, our results confirmed the association between brand prestige and WBP was strengthened for the seniors who perceived high tour guide services. The salient role of tour guide services was indicated through many studies [7,10,55], and our analysis results provided the empirical evidences in the senior tourism. Thus, first and foremost, unceasing efforts should be made to improve the tour guides services of senior tour packages. Tsaur and Teng [10] emphasized the senior tourists’ heavy reliance on tour guide due to their barriers and difficulties and asserted tour guide must adopt his/her style to provide additional care and greater concern. Furthermore, SERT [80] explained the greater needs of senior tourists according to characteristics, lifestyle or habits. In this regard, extensive training and developing programs should be established in a way that tour guide can gain a high level of knowledge of seniors, easily flex their styles to deal with different types of seniors, and practice their services in many fields. Tour guide services are not limited to the instrumental role but encompass many others such as interactional and communicative roles [10]. Therefore, the programs with a variety of different situations and profiles of seniors (e.g., age, gender, nationality, or lifestyle) should be well designed and prepared for tour operators to ensure tour guides have a high level of competence and consequently improve seniors’ WBP. Meanwhile, collaborations with local experts can be considered as the part of tour guide services. For instance, sessions hosted by local historians at a historical site may upgrade tour guide services as it provides vivid storytelling and more authentic services.

Fifth, the result from the multiple-group analysis provided evidence regarding the significant moderating impact of tour guide services in the relation between consumer attitude and WOM. In particular, the magnitude of the relationship between these two variables (*β*_low group_ = 0.149, *p* < 0.05; *β*_high group_ = 0.515, *p* < 0.05) was significantly greater in the group of people who perceived the high tour guide services. This result is consistent with prior research [9,82] and implies that at a similar level of senior tourists’ attitude, customers who highly appreciate tour guide services more actively build a favorable WOM than customers who do not or appreciate less. Hence, tactical approaches are recommended to spread more positive words from the seniors who possess better views of tour guide services. For example, it is first needed to identify the group of people who recognized a high level of tour guide services, and this can be managed through the satisfaction survey right after the package tour. Then, the encouragement of WOM to the identified specific group of people should be arranged. In order to make it further effective, a small souvenir can be utilized as a gesture of appreciation and value added (e.g., upgrading the tour programs, free yoga class, or menu upgrade) or discounts can be extended to the friends who were introduced by their WOM. On the other hand, a follow up for the group indicating less satisfaction on tour guide services should be arranged to avoid any negative or neutral WOM transmittance, which are unnecessary. 

## 6. Limitations and Future Research

First, the current study collected data from elderly tourists in South Korea, so it is somewhat difficult to apply the findings of this study to other countries. Similarly, this study focused on the senior tourism industry, so there are limitations in applying the findings of this study to other fields. Second, our study dealt with senior tourists as individuals over 65 years old following the legal standard in South Korea. However, there is no consensus defining senior tourists by chronological age around the world; a broad scope was proposed that included health, retirement, socio-economic circumstances, subjective age, or income levels [78,81]. Thus, other important parameters shaping the senior tourists are recommended for studies in future. Third, the current paper employed a nonprobability convenience-sampling approach. Even though this method is a widely known method in the tourism industry, it is difficult to represent the entire population. Hence, future studies are necessary to use greater sampling range. Finally, as a result of data collection, there were more than twice as many females as males, so gender distribution was uneven. Future research, however, requires data collection that takes gender ratios into account.

## 7. Conclusions

An increase of proportion of senior tourists would bring new opportunities in the tourism sector [78,79]. In particular, because senior tourists prefer the organized tours to ensure a safe journey and the immediate assistance for any unforeseen problems during travel, the growing interest is reflected in tour packages, which are excursions or holidays in a group led by a tour guide with a combination of various services [10,80]. On the other hand, WBP has been spotlighted in the tourism context as more tourists are concerned with the QOL and this equally applies to the senior tourism industry [5,6]. Despite the tremendous opportunities, namely, growing demands, in the senior tourism and greater interest in wellbeing, the existing literature in the domain of the tourism industry has offered a limited view pertinent to the seniors’ WBP in associations with brand prestige and its outcomes. That is to say, little is known about the intricate relationships among brand prestige, WEP, attitude, and WOM. In addition, no attempt was made to investigate the moderating effect of tour guide services in the links among these key variables in the elderly tourism context. The present study successfully filled this void as well as considering the roles of tour guide services in such relationships. The findings accordingly contribute to offering meaningful implications in academia and industry. 

## Figures and Tables

**Table 1:** Profile of survey respondents (*n* = 325).

Variable	*N*	Percentage
Gender		
Men	95	29.2
Women	228	70.2
Educational Level		
High school diploma	73	22.5
Associate’s degree	29	8.9
Bachelor’s degree	181	55.7
Graduate degree	42	12.9
Marital Status		
Single	4	1.2
Married	314	96.6
Others (divorced and widow/widower)	7	2.2
Monthly income		
Less than US$ 1000	8	2.5
US$ 1001~US$ 2000	54	16.6
US$ 2001~US$ 3000	58	17.8
US$ 3001~US$ 4000	71	21.8
US$ 4001~US$ 5000	59	18.2
US$ 5001~US$ 6000	47	14.5
More than US$ 6001	28	8.6
Mean age = 69.20 years old		

**Table 2:** Confirmatory factor analysis: Items and loadings.

Construct and Scale Item	Standardized Loading ^a^
Package tour prestige	
My package tour is prestigious.	0.935
My package tour has high status.	0.940
My package tour is very upscale.	0.897
Seniors’ WBP	
My package tour plays an important role in my well-being.	0.925
My package tour meets my overall well-being needs.	0.938
My package tour plays an important role in enhancing my QOL.	0.918
Consumer attitude	
Favorable—Unfavorable	0.899
Like—Dislike	0.916
Good—Bad	0.918
Pleasant—Unpleasant	0.924
Positive—Negative	0.922
WOM	
I will encourage others to use the travel agency.	0.907
I will spread the news about the good aspects of the travel agency to others.	0.966
I will recommend the travel agency to others.	0.926
Goodness-of-fit statistics: χ^2^ = 259.722, *df* = 71, *p* < 0.001, χ^2^/*df* = 3.658, NFI = 0.957, CFI = 0.968, IFI = 0.968, TLI = 0.959, RMSEA = 0.071	

Notes 1: ^a^ All factors loadings are significant at *p* < 0.001. Notes 2: NFI = Normed Fit Index, CFI = Comparative Fit Index, IFI = Incremental Fit Index, TLI = Tucker-Lewis Index, RMSEA = Root Mean Square Error of Approximation.

**Table 3:** Descriptive statistics and associated measures.

Variables	Mean (SD)	AVE	(1)	(2)	(3)	(4)
(1) Brand prestige	4.30 (0.94)	0.854	**0.946 ^a^**	0.716 ^b^	0.746	0.677
(2) Seniors’ WBP	4.37 (0.95)	0.859	0.513^c^	**0.948**	0.750	0.696
(3) Consumer attitude	4.52 (1.10)	0.839	0.557	0.563	**0.963**	0.733
(4) WOM	4.40 (0.99)	0.871	0.458	0.484	0.537	**0.953**

Notes 1: SD = Standard Deviation, AVE = Average Variance Extracted. Notes 2: ^a^ Composite reliabilities are along the diagonal, ^b^ Correlations are above the diagonal, and ^c^ Squared correlations are below the diagonal.

**Table 4:** Standardized parameter estimates for structural model.

Independent Variable		Dependent Variable	Beta	*t*-Value	Result
H1: Brand prestige	→	Seniors’ WBP	0.896	21.236	Supported
H2: Brand prestige	→	Consumer attitude	0.371	3.503	Supported
H3: Brand prestige	→	WOM	0.102	0.939	Not supported
H4: Seniors’ WBP	→	Consumer attitude	0.418	3.941	Supported
H5: Seniors’ WBP	→	WOM	0.255	2.351	Supported
H6: Consumer attitude	→	WOM	0.465	7.097	Supported
H7a: The moderating of tour guide services in the relationship between brand prestige and seniors’ WBP					Supported
H7b: The moderating of tour guide services in the relationship between brand prestige and consumer attitude					Not supported
H7d: The moderating of tour guide services in the relationship between seniors’ WBP and consumer attitude					Not supported
H7e: The moderating of tour guide services in the relationship between seniors’ WBP and WOM					Not supported
H7f: The moderating of tour guide services in the relationship between consumer attitude and WOM					Supported
Goodness-of-fit statistics: χ^2^ = 259.722, *df* = 71, *p* < 0.001, χ^2^/*df* = 3.658, NFI = 0.957, CFI = 0.968, IFI = 0.968, TLI = 0.959, RMSEA = 0.070					

Notes 1: *p* < 0.05. Notes 2: SE = Standardized Estimate. Notes 3: NFI = Normed Fit Index, CFI = Comparative Fit Index, IFI = Incremental Fit Index, TLI = Tucker-Lewis Index, RMSEA = Root Mean Square Error of Approximation.
